# First detection and genetic characterization of canine Kobuvirus in domestic dogs in Thailand

**DOI:** 10.1186/s12917-019-1994-6

**Published:** 2019-07-19

**Authors:** Kamonpan Charoenkul, Taveesak Janetanakit, Supassama Chaiyawong, Napawan Bunpapong, Supanat Boonyapisitsopa, Ratanaporn Tangwangvivat, Alongkorn Amonsin

**Affiliations:** 1 Center of Excellence for Emerging and Re-emerging Infectious Diseases in Animals, Faculty of Veterinary Science, Chulalongkorn University, Bangkok, Thailand; 2 Department of Veterinary Public Health, Faculty of Veterinary Science, Chulalongkorn University, Bangkok, 10330 Thailand; 3 Veterinary Diagnostic Laboratory, Faculty of Veterinary Science, Chulalongkorn University, Bangkok, Thailand

**Keywords:** Canine, Characterization, Detection, Kobuvirus, Thailand

## Abstract

**Background:**

Canine Kobuvirus (CaKoV) has been detected both in healthy and diarrheic dogs and in asymptomatic wild carnivores. In this study, we conducted a survey of CaKoV at small animal hospitals in Bangkok and vicinity of Thailand during September 2016 to September 2018.

**Results:**

Three hundred and seven rectal swab samples were collected from healthy dogs (*n* = 55) and dogs with gastroenteritis symptoms (*n* = 252). Of 307 swab samples tested by using one-step RT-PCR specific to 3D gene, we found CaKoV positivity at 17.59% (54/307). CaKoVs could be detected in both sick (19.44%) and healthy (9.09%) animals. In relation to age group, CaKoV could be frequently detected in younger dogs (25.45%). Our result showed no seasonal pattern of CaKoV infection in domestic dogs. In this study, we characterized CaKoVs by whole genome sequencing (*n* = 4) or 3D and VP1 gene sequencing (*n* = 8). Genetic and phylogenetic analyses showed that whole genomes of Thai CaKoVs were closely related to Chinese CaKoVs with highest 99.5% amino acid identity suggesting possible origin of CaKoVs in Thailand.

**Conclusions:**

In conclusion, this study was the first to report the detection and genetic characteristics of CaKoVs in domestic dogs in Thailand. CaKoVs could be detected in both sick and healthy dogs. The virus is frequently detected in younger dogs. Thai CaKoVs were genetically closely related and grouped with Chinese CaKoVs. Our result raises the concerns to vet practitioners that diarrhea in dogs due to canine Kobuvirus infection should not be ignored.

**Electronic supplementary material:**

The online version of this article (10.1186/s12917-019-1994-6) contains supplementary material, which is available to authorized users.

## Background

Kobuvirus (KoV) is a single-strand positive-sense RNA virus**.** KoV belongs to the family Piconaviridae, genus Kobuvirus, which consists of four species Aichivirus A, B, C and D [1–3]. KoV has been reported in feces of several mammal species including humans, ruminants, pigs, dogs, cats, bats and rodents [3–10]. The Kobuvirus species Aichivirus A contains four types including Aichi virus 1, canine Kobuvirus 1 (CaKoV), Feline Kobuvirus 1 (FeKoV) and Murine Kobuvirus 1 (MuKoV). Canine Kobuvirus 1 (CaKoV) was first reported in dogs with acute gastroenteritis in the US in 2011 [5, 11]. CaKoV was subsequently reported in dogs in UK, Italy, Australia, Japan, Korea and China [4, 12–15]. The virus was reported in wild carnivores (Jackal and Hyena) and domestic dogs in Tanzania, Africa [16], in foxes in Spain [17] and in foxes [18] and wolves in Italy [19]. Several studies have reported the detection of CaKoV infection in dogs with or without diarrhea and sometime systemic infection [20]. To date, only 12 completed CaKoV genomes are available in the GenBank database.

During September 2016 to September 2018, the center of excellence for emerging and re-emerging infectious diseases in animals (CUEIDAs), Chulalongkorn University conducted a survey of canine Kobuvirus in domestic dogs at small animal hospitals in 5 provinces of Thailand. The survey was conducted under the Chulalongkorn University’s animal use and care protocol # 1731074. The result of this study provided the first detection and genetic characterization of CaKoV isolated from domestic dogs in Thailand.

## Results

### Canine Kobuviruses in domestic dogs in Thailand

**Fig. 1:**
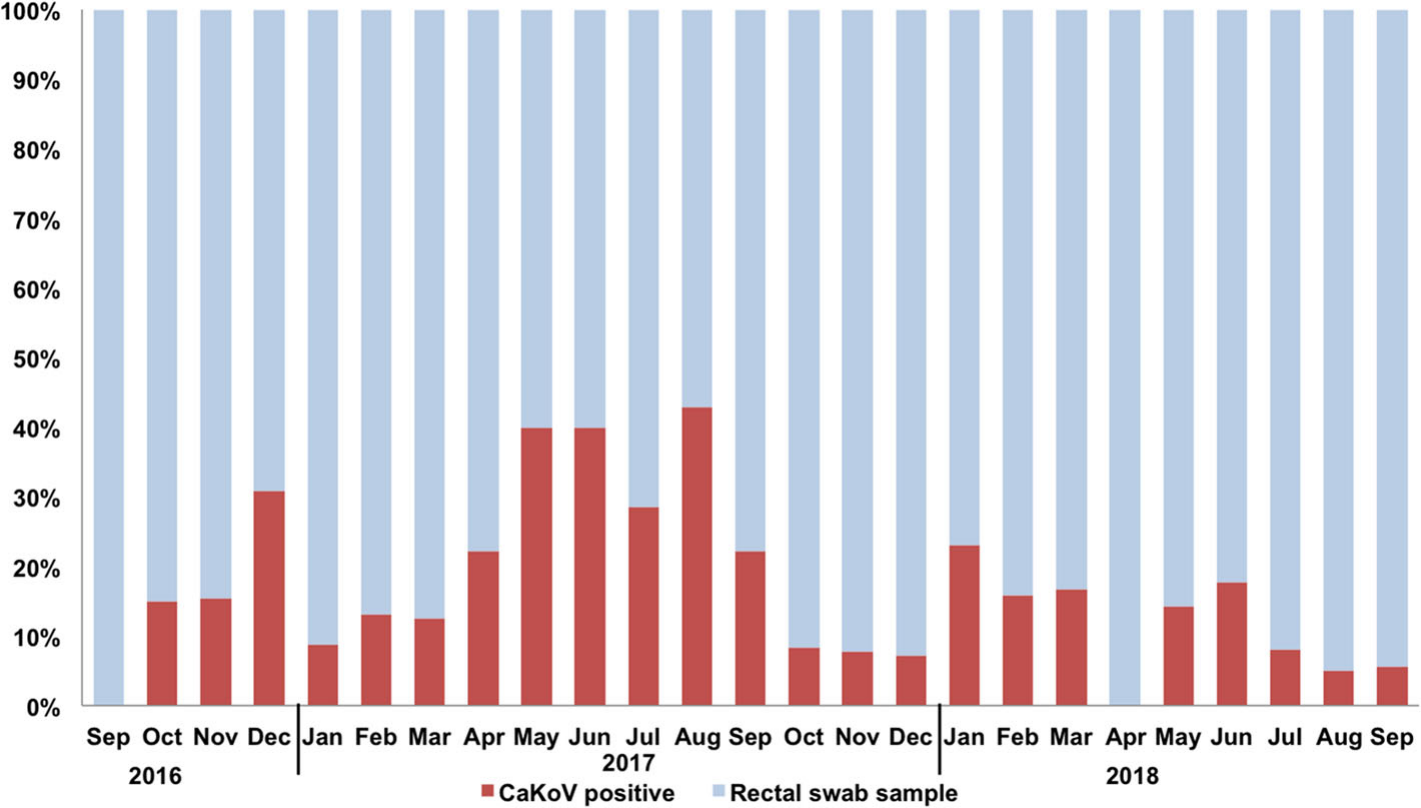
Number of samples collected and CaKoVs detected by month in this study

**Fig. 2:**
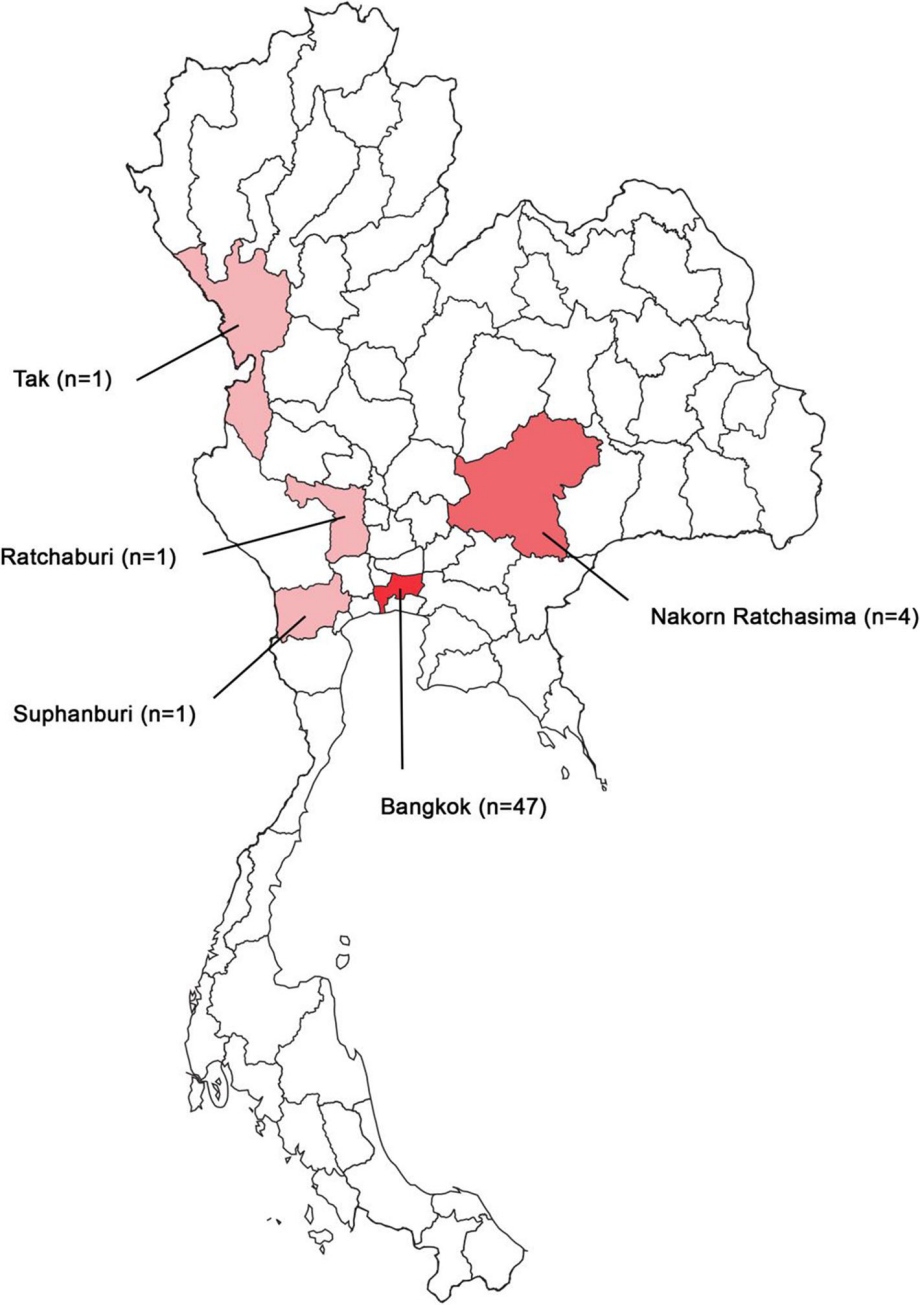
Number of samples collected and CaKoVs detected by provinces in this study (Map of Thailand with permission by World Trade Press)

During September 2016 to September 2018, we conducted a survey of viral enteric diseases in domestic dogs in small animal hospitals in 5 provinces of Thailand (Bangkok, Nakhon Ratchasima, Ratchaburi, Suphanburi, and Tak). We tested 307 rectal swab samples for CaKoV by using one-step RT-PCR specific to 3D gene. Based on a two-year survey, we found CaKoV positivity at 17.59% (54/307). CaKoVs could be detected in both sick (19.44% (49/252)) and healthy (9.09% (5/55)) animals. Our result showed no seasonal pattern of CaKoV infection in dogs (Figs. 1 and 2). In relation to age group, CaKoV could be frequently detected in younger dogs at 25.45% (42/165) (Additional file 2: Table S2). The co-infections of CaKoV with other enteric viral pathogens were observed including CaKoV/Canine parvovirus/Canine Coronavirus (*n* = 6), CaKoV/Canine parvovirus (*n* = 20) and CaKoV/Canine Coronavirus (*n* = 2). In this study, 12 CaKoVs were selected and characterized by whole genome sequencing (*n* = 4) or 3D and VP1 gene sequencing (*n* = 8). The viruses were selected to represent epidemiological and demographic data such as age, date of isolation and breed. In this study, nucleotide sequences of the CaKoV were submitted to the GenBank database under the accession numbers MK201776 - MK201795 (Table 1).

**Table 1:** Detail description of Thai CaKoVs characterized in this study

Virus	Date	Location	Region	Age	Breed	Clinical signs	Sequencing	GenBank Accession number
CU-53	Oct-16	Bangkok	Central	2 months	Pomeranian	Diarrhea	WG^a^	MK201776
CU-101	Dec-16	Bangkok	Central	3 months	Pekingese	Diarrhea	WG	MK201777
CU-249	May-17	Bangkok	Central	3 months	Pomeranian	Diarrhea	WG	MK201778
CU-716	Jan-18	Bangkok	Central	12 years	Shizu	Diarrhea	WG	MK201779
CU-83	Nov-16	Bangkok	Central	2 months	Pomeranian	Diarrhea	3D, VP1^b^	MK201780, MK201788
CU-100	Dec-16	Ratchaburi	Central	6 months	Great Dane	Diarrhea	3D, VP1	MK201781, MK201789
CU-125	Jan-17	Tak	Northern	2 months	Bang Keaw	Asymptomatic	3D, VP1	MK201782, MK201790
CU-224	Feb-17	Bangkok	Central	9 years	Pomeranian	Diarrhea	3D, VP1	MK201783, MK201791
CU-241	Apr-17	Bangkok	Central	3 months	Mixed	Diarrhea	3D, VP1	MK201784, MK201792
CU-250	May-17	Bangkok	Central	3 months	Pomeranian	Diarrhea	3D, VP1	MK201785, MK201793
CU-260	Jun-17	Nakhon Ratchasima	North- Eastern	2 months	German Shepherd	Diarrhea	3D, VP1	MK201786, MK201794
CU-273	Aug-17	Bangkok	Central	2 months	Pomeranian	Diarrhea	3D, VP1	MK201787, MK201795

^a^*WG* Whole genome sequencing

^b^ 3D, VP1: 3D and VP1 gene sequencing

### Phylogeny of the Thai canine Kobuviruses

**Fig. 3:**
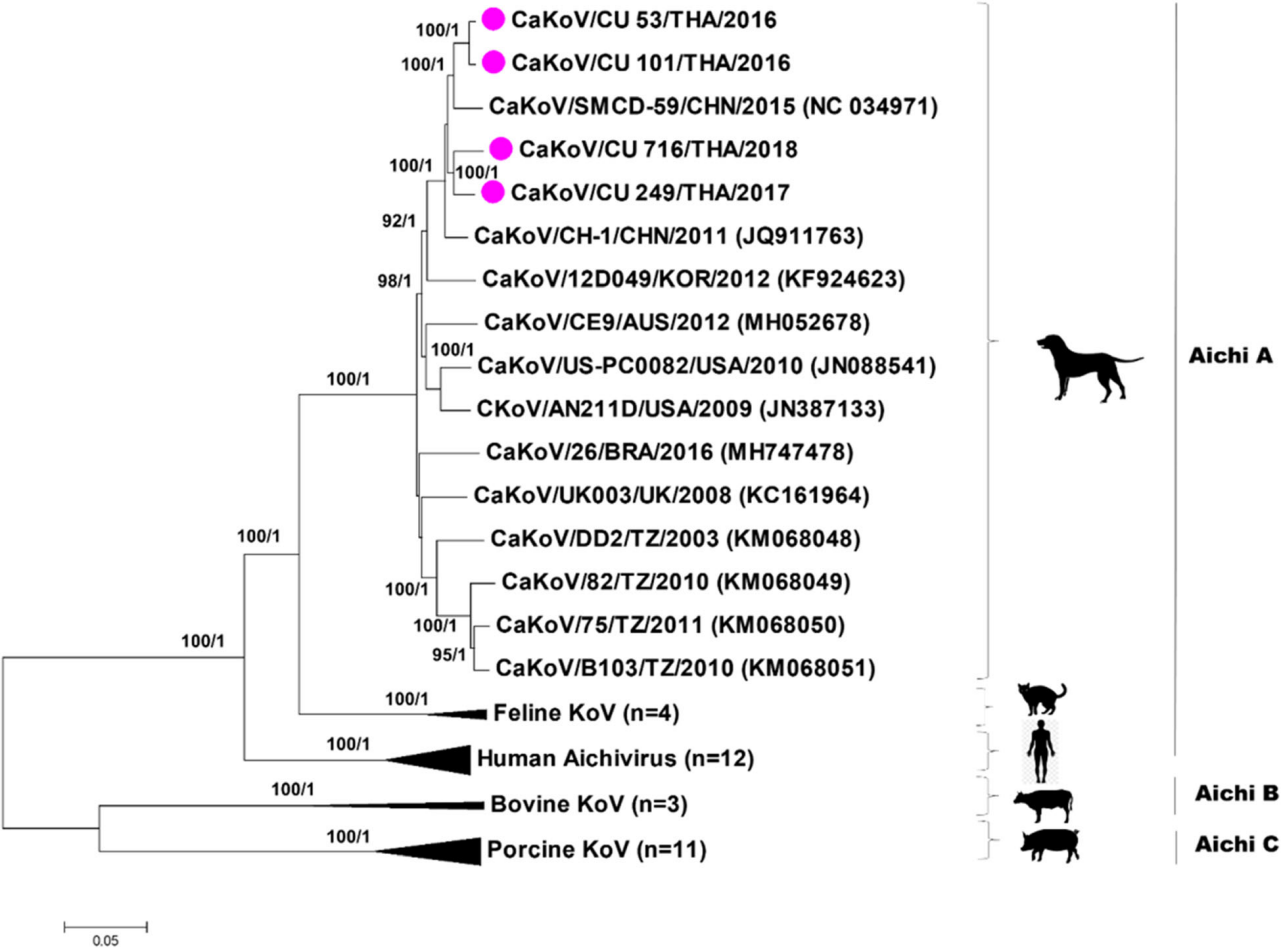
Phylogenic tree of the completed genome of CaKoVs. The phylogenetic tree was constructed by using MEGA v6.0 with neighbor-joining algorithm with Kimura-2 parameter model and Beast program with Bayesian Markov chain Monte Carlo (BMCMC) with 10,000,000 generations and an average standard deviation of split frequencies < 0.05. Values on branches represent bootstrap and posterior probability values

**Fig. 4:**
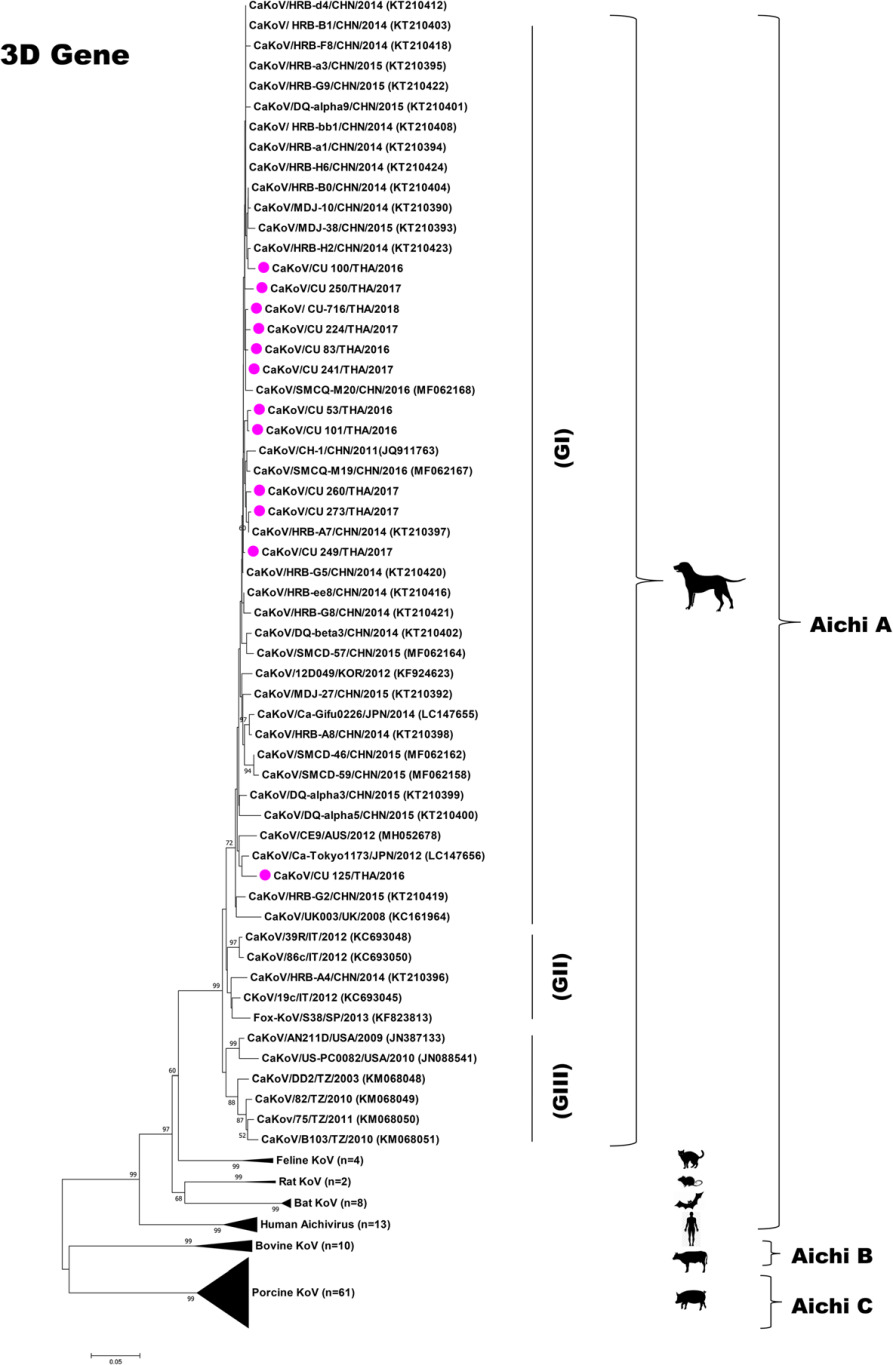
Phylogenetic tree of 3D gene of CaKoVs. The phylogenetic tree was constructed by using MEGA v6.0 with neighbor-joining algorithm with Kimura-2 parameter model with 1,000 replications of bootstrap analysis

**Fig. 5:**
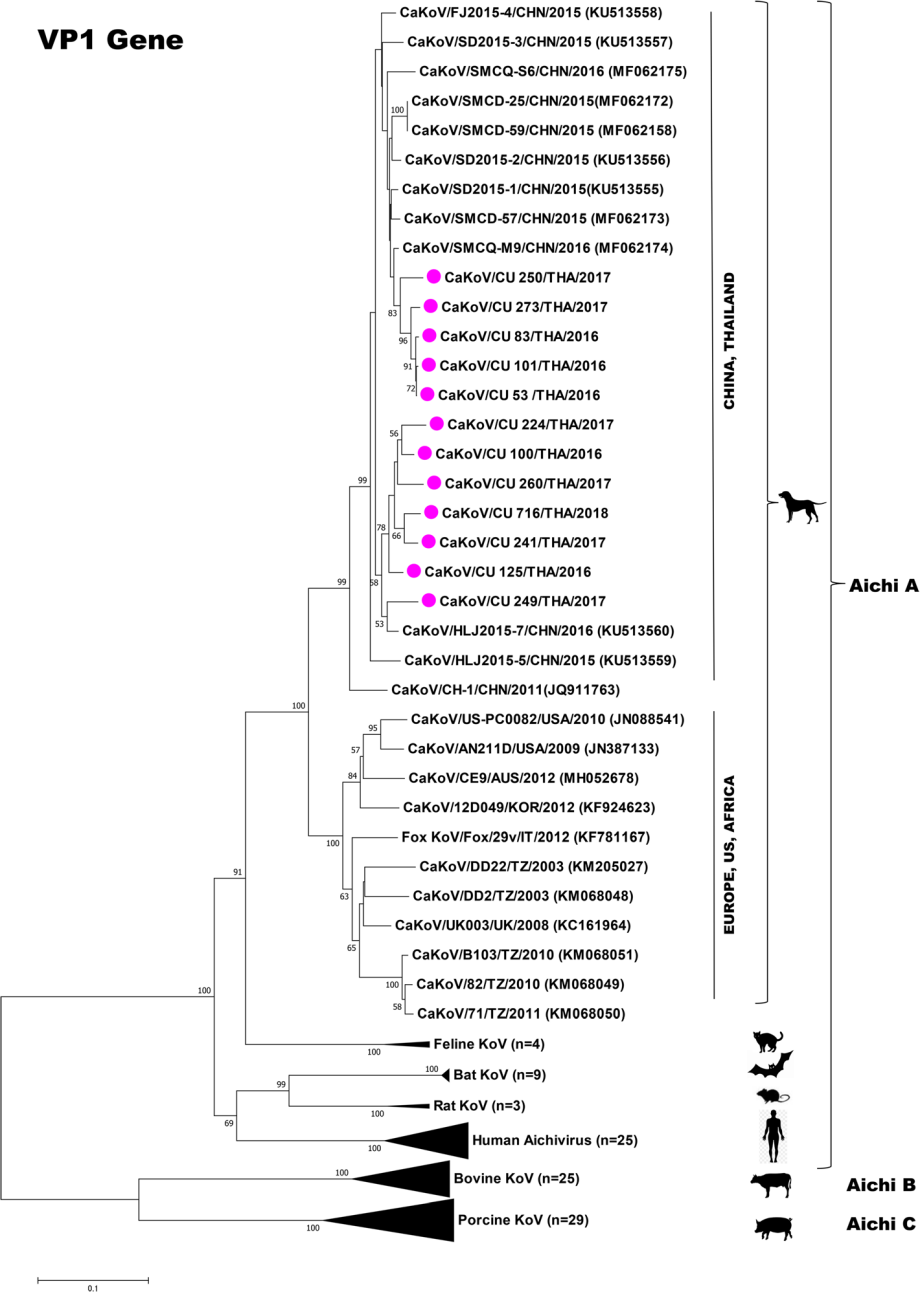
Phylogenetic tree of VP1 gene of CaKoVs. The phylogenetic tree was constructed by using MEGA v6.0 with neighbor-joining algorithm with Kimura-2 parameter model with 1,000 replications of bootstrap analysis

Phylogenetic analysis of whole genome of CaKoVs showed that the Thai CaKoVs were closely related to each other and clustered with Aichivirus A. The cluster Aichivirus A contains Kobuviruses from dogs, cats, rodents, bats and human. While Aichivirus B and C contain Kobuviruses from cattle and pigs, respectively. Based on whole genome sequence, Thai CaKoVs were closely related to Chinese CaKoVs sub-cluster but in separated sub-cluster from the viruses from the US, UK, Brazil and Tanzania (Fig. 3). Phylogenetic analysis of 3D and VP1 of Thai CaKoVs and reference CaKoVs from various animal species were also performed. Similarly, 3D gene of Thai CaKoVs were grouped together with Chinese CaKoVs (G1 sub-cluster) but separated from the viruses in sub-clusters G2 as well as G3 (Fig. 4). Phylogenetic analysis of VP1 gene, the viruses can be clustered into 2 major subgroups, US/EU/Africa subgroup and China/Thailand subgroup (Fig. 5).

### Genetic analysis of the Thai canine Kobuviruses

We compared the nucleotide and deduced amino acid sequences of Thai CaKoVs against those of reference viruses from the US, UK, Italy, China, and Korea (Tables 2 and 3). Our results showed that whole genome of 4 Thai CaKoVs (CU-53, CU-101, CU-249 and CU-716) shared 96.7–99.3% nucleotide similarity (99.6–100% amino acid similarity) to each other and posed highest nucleotide similarity to Chinese CaKoVs including SMCD-59 (97.0% nt and 99.5% aa identity) and CH-1 (96.8% nt and 98.7% aa identity). Our analysis showed that the VP1 protein was the most diverse gene with 93.4–99.9% nucleotide similarity (96.9–100% aa similarity) among Thai CaKoVs and 82.2–96.8% with other reference CaKoVs. The most variable region of VP1 is position 201–243, especially proline rich region. Putative proline rich region at VP1–228-240 (P_228_XPPPPXPPXPXP_240_) was also observed in Thai CaKoVs as well as reference viruses (Table 4). In this study, unique amino acids were found in Thai and Chinese CaKoVs at the position, 65 V, 67D, 119L, 138T, 150P, 151M, 153D, 201S, 204Q, 205Q, 201Q, 213T and 241E (Table 4). Analysis of predicted amino acid cleavage sits of whole genome were conserved among Thai CaKoVs (Table 5).

**Table 2:** Pairwise comparison of whole genome of Thai CaKoVs (CU-101) and reference CaKoVs

				% nucleotide identity (% amino acid identity)										
Virus	Accession number	Year	Country	WGS	VP0	VP3	VP1	2A	2B	2C	3A	3B	3C	3D
CaKoV/CU-101/THA/2016	This study	2016	Thailand	100 (100)	100 (100)	100 (100)	100 (100)	100 (100)	100 (100)	100 (100)	100 (100)	100 (100)	100 (100)	100 (100)
CaKoV/CU-53/THA/2016	This study	2016	Thailand	99.3 (100)	99.8 (100)	99.3 (100)	99.5 (100)	100 (100)	97.1 (100)	99.4 (100)	100 (100)	100 (100)	100 (100)	98.8 (100)
CaKoV/CU-249/THA/2017	This study	2017	Thailand	96.7 (99.6)	96.2 (100)	97.2 (99.6)	94.3 (98.2)	100 (100)	97 (100)	97.2 (100)	97.2 (98.9)	96.3 (100)	97.6 (99.7)	97.5 (100)
CaKoV/CU-716 /THA/2018	This study	2018	Thailand	96.7 (99.8)	95.2 (100)	97.5 (99.6)	95.2 (99.6)	100 (100)	96 (100)	96.8 (100)	96.8 (98.9)	96.3 (96.3)	98.3 (99.7)	97.5 (100)
CaKoV/CH-1/CHN/2011	JQ911763	2011	China	96.8 (98.7)	97.4 (99.7)	97.6 (99.6)	91.2 (91.8)	98.3 (100)	97.1 (100)	97.3 (99.7)	98.6 (100)	100 (100)	98.5 (99.7)	97.1 (100)
CaKoV/SMCD-59/CHN/2015	NC034971	2015	China	97 (99.5)	92.5 (98.7)	93 (99.6)	96.5 (97.8)	100 (100)	95.3 (100)	96 (100)	94.7 (97.9)	89 (96.3)	95.6 (99.2)	95.3 (99.6)
CaKoV/12D049/KOR/2012	KF924623	2012	Korea	94.2 (97.9)	93.2 (98.7)	93.2 (99.6)	85.5 (90)	100 (100)	97.3 (100)	96.4 (99.1)	97.2 (98.9)	93.9 (96.3)	96.9 (98.7)	94.7 (98.5)
CaKoV/U\K003/UK/2008	KC161964	2008	UK	93.6 (98.1)	92.4 (99)	95.2 (99.6)	86 (89.6)	100 (100)	93.4 (99)	95.3 (99.7)	94.3 (98.9)	91.5 (96.3)	95.9 (99.2)	96.4 (100)
CaKoV/26/BRA/2016	MH747478	2016	Brazil	92.8 (97.9)	91.1 (99)	91.7 (99.1)	83.2 (86)	100 (100)	95.6 (100)	95.8 (100)	96.5 (98.9)	87.8 (96.3)	94.7 (98.2)	96.5 (99.6)
CaKoV/US-PC0082/USA/2010	JN088541	2010	USA	93.4 (97.7)	91.1 (97.4)	92.3 (99.1)	85.7 (88.2)	100 (100)	93.3 (98)	94.2 (99.4)	92.9 (98.9)	89 (96.3)	94.6 (99)	94.4 (98.9)
CaKoV/CE9/AUS/2012	MH052678	2012	Australia	93.7 (97.6)	97.6 (99.5)	96.4 (100)	85.7 (90)	100 (100)	97.6 (100)	97.6 (100)	96.8 (98.9)	95.1 (100)	97.4 (99.5)	97 (100)
CaKov/75/TZ/2011	KM068050	2011	African	92.1 (97.5)	92.5 (99.2)	92.4 (99.6)	84.2 (88.5)	100 (100)	96.3 (99.5)	95.6 (99.4)	95 (98.9)	90.2 (96.3)	96.4 (99.2)	96.5 (99.6)
CaKoV/B103/TZ/2010	KM068051	2010	African	92.2 (97.5)	90.8 (96.9)	91.8 (98.7)	84.7 (89.2)	100 (100)	93.4 (99)	93.8 (99.4)	92.9 (98.9)	89 (96.3)	94.6 (99.2)	94.9(99.3)
CaKoV/DD2/TZ/2003	KM068048	2003	African	92.3 (97.9)	91 (98.7)	94.1 (99.6)	84.3 (89.2)	100 (100)	93.4 (99)	93.2 (98.8)	92.6 (98.9)	89 (96.3)	94.9 (99.2)	94.9 (99.6)
CaKoV/82/TZ/2010	KM068049	2010	African	91.8 (96.5)	91 (97.1)	92.4 (99.1)	84.2 (87.8)	100 (100)	93.3 (98)	92.8 (98.8)	92.2 (97.9)	91.5 (96.3)	94.4 (98.7)	94.4 (98.9)

**Table 3:** Pairwise comparison of 3D and VP1 genes of Thai CaKoVs (CU-101) and reference CaKoVs

Viruses	Accession number	Year	Country	% nucleotide identity (% amino acid identity)	
				3D	VP1
CaKoV/CU-101/THA/2016	This study	2016	Thailand	100 (100)	100 (100)
CaKoV/CU-53/THA/2016	This study	2016	Thailand	99.5 (100)	99.9 (100)
CaKoV/CU-83/THA/2016	This study	2016	Thailand	98.8 (100)	99.7 (100)
CaKoV/CU-100/THA/2016	This study	2016	Thailand	97.9 (100)	93.6 (97.8)
CaKoV/CU-125/THA/2016	This study	2016	Thailand	97.1 (98.6)	94.9 (97.8)
CaKoV/CU-224/THA/2017	This study	2017	Thailand	98.6 (100)	93.6 (97.8)
CaKoV/CU-241/THA/2017	This study	2017	Thailand	99.0 (100)	94.5 (98.7)
CaKoV/CU-249/THA/2017	This study	2017	Thailand	98.8 (100)	93.6 (97.4)
CaKoV/CU-250/THA/2017	This study	2017	Thailand	98.1 (100)	96.6 (96.9)
CaKoV/CU-260/THA/2017	This study	2017	Thailand	98.6 (100)	93.4 (96.9)
CaKoV/CU-273/THA/2017	This study	2017	Thailand	98.6 (100)	98.5 (99.1)
CaKoV/CU-716/THA/2018	This study	2018	Thailand	98.8 (100)	94.3 (98.7)
CaKoV/26/BRA/2016	MH747478	2016	Brazil	97.1 (99.3)	82.2 (84.2)
CaKoV/CE9/AUS/2012	MH052678	2012	Australia	97.6 (100)	83.7 (87.7)
CaKoV/B103/TZ/2010	KM068051	2010	African	93.6 (98.6)	84.8 (88.2)
CaKov/75/TZ/2011	KM068050	2011	African	94.0 (97.9)	83.8 (86.4)
CaKoV/82/TZ/2010	KM068049	2010	African	94.5 (98.6)	84.3 (86.4)
CaKoV/DD2/TZ/2003	KM068048	2003	African	94.8 (99.3)	84.0 (87.7)
CaKoV/UK003/UK/2008	KC161964	2008	UK	96.0 (100)	85.3 (88.2)
CaKoV/US-PC0082/USA/2010	JN088541	2010	USA	94.0 (99.3)	84.5 (86.4)
CaKoV/AN211D/USA/2009	JN387133	2009	USA	95.2 (99.3)	84.4 (86.8)
CaKoV/86c/IT/2012	KC693050	2012	Italy	96.0 (99.3)	N/A
CKoV/19c/IT/2012	KC693045	2012	Italy	96.2 (99.3)	N/A
CaKoV/Ca-Gifu0226/JPN/2014	LC147655	2014	Japan	97.6 (99.3)	N/A
CaKoV/Ca-Tokyo1173/JPN/2012	LC147656	2012	Japan	97.9 (100)	N/A
CaKoV/12D049/KOR/2012	KF924623	2012	Korea	97.1 (100)	84.7 (89.0)
CaKoV/CH-1/CHN/2011	JQ911763	2016	China	97.9 (100)	91.3 (89.9)
CaKoV/SMCD-59/CHN/2015	MF062158	2015	China	97.1 (100)	96.4 (96.9)
CaKoV/SMCD-57/CHN/2015	MF062173	2015	China	97.9 (100)	96.8 (97.8)

**Table 4:** Genetic analysis of Thai CaKoVs compared with reference CaKoVs at proline rich region

Viruses	Accession number	Year	Country	Amino acid at position													
				65	67	119	138	150	151	153	201	204	205	210	213	241	Proline rich region (228–240)
CaKoV/CU-101/THA/2016	This study	2016	Thailand	V	D	L	T	P	M	D	S	Q	Q	Q	T	E	PRAPPPLPPLPTP
CaKoV/CU-53/THA/2016	This study	2016	Thailand	V	D	L	T	P	M	D	S	Q	Q	Q	T	E	PRAPPPLPPLPTP
CaKoV/CU-249/THA/2017	This study	2017	Thailand	V	D	L	T	P	M	D	S	Q	Q	Q	T	E	PRAPPPLPPLPTP
CaKoV/CU-716/THA/2018	This study	2018	Thailand	V	D	L	T	P	M	D	S	Q	Q	Q	T	E	PRAPPPLPPLPTP
CaKoV/SMCQ-M9/CHN/2016	MF062174	2016	China	V	D	L	T	P	M	D	S	Q	Q	Q	T	E	PRAPPPLPPLPTP
CaKoV/SMCD-59/CHN/2015	NC 034971	2015	China	V	D	L	T	P	M	D	S	Q	Q	Q	T	E	PRAPPPLPPLPTP
CaKoV/12D049/KOR/2012	KF924623	2012	Korea	L	N	V	M	S	E	N	T	V	E	S	S	A	PRAPPPLPPLPTP
CaKoV/CE9/AUS/2012	MH052678	2012	Australia	L	N	V	M	S	E	N	T	V	E	S	S	T	PRAPP-LPPLPTP
CaKoV/AN211D/USA/2009	JN387133	2009	USA	L	N	P	M	S	E	N	T	V	E	S	S	A	PRAPPPLPPLPTP
CaKoV/US-PC0082/USA/2010	JN088541	2010	USA	L	N	V	M	S	E	N	T	V	E	S	S	A	CPVPPPLPPLPTP
CaKoV/UK003/UK/2008	KC161964	2008	UK	L	N	V	M	S	E	N	T	V	E	S	S	T	PRAPPPLPPLPTP
CaKoV/26/BRA/2016	MH747478	2016	Brazil	L	N	V	M	S	E	N	T	V	E	S	S	T	HGAPPPLPPLPTP
CaKoV/75/TZ/2011	KM068050	2011	Africa	L	N	V	M	S	E	N	T	A	E	S	S	T	CPVPPPLPPLPTP
CaKoV/82/TZ/2010	KM068049	2010	Africa	L	N	V	M	S	E	N	T	A	E	S	S	T	CPVPPPLPPLPTP
CaKoV/B103/TZ/2010	KM068051	2010	Africa	L	N	V	M	S	E	N	T	A	E	S	S	T	PRAPPPLPPLPTP
CaKoV/DD2/TZ/2003	KM068048	2003	Africa	L	N	V	M	S	E	N	T	V	E	S	S	T	PRAPPPLPPLPTP

**Table 5:** Genetic analysis of Thai CaKoVs compared with reference CaKoVs at putative amino acid cleavage sites

Viruses	Year	Country	Amino acid position									
			171/172	553/554	776/777	1054/1055	1165/1166	1330/1331	1665/1666	1759/1760	1786/1787	2176/2177
CU-53	2016	Thailand	Q/G	Q/H	Q/A	Y/V	Q/G	Q/G	Q/G	Q/A	Q/G	Q/G
CU-101	2016	Thailand	Q/G	Q/H	Q/A	Y/V	Q/G	Q/G	Q/G	Q/A	Q/G	Q/G
CU-249	2017	Thailand	Q/G	Q/H	Q/A	Y/V	Q/G	Q/G	Q/G	Q/A	Q/G	Q/G
CU-716	2018	Thailand	Q/G	Q/H	Q/A	Y/V	Q/G	Q/G	Q/G	Q/A	Q/G	Q/G
12D049	2012	Korea	Q/G	Q/H	Q/A	Y/V	Q/G	Q/G	Q/G	Q/A	Q/G	Q/G
UK003	2008	UK	Q/G	Q/H	Q/A	Y/V	Q/G	Q/G	Q/G	Q/A	Q/G	Q/G
26/BRA	2016	Brazil	Q/G	Q/H	Q/A	Y/V	Q/G	Q/G	Q/G	Q/A	Q/G	Q/G
SMCD-59	2015	China	Q/G	Q/H	Q/A	Y/V	Q/G	Q/G	Q/G	Q/A	Q/G	Q/G
CE9	2012	Australia	Q/G	Q/H	Q/A	Y/V	Q/G	Q/G	Q/G	Q/A	Q/G	Q/G
B103	2010	Africa	Q/G	Q/H	Q/T ^a^	Y/V	Q/G	Q/G	Q/G	Q/A	Q/G	Q/G
75	2011	Africa	Q/G	Q/H	Q/A	Y/V	Q/G	Q/G	Q/G	Q/A	Q/G	Q/G
82	2010	Africa	Q/G	Q/H	Q/T ^a^	Y/V	Q/G	Q/G	Q/G	Q/A	Q/G	Q/G
DD2	2003	Africa	Q/G	Q/H	Q/A	Y/V	Q/G	Q/G	Q/G	Q/A	Q/G	Q/G
US-PC0082	2010	USA	Q/G	Q/H	Q/A	Y/V	Q/G	Q/G	Q/G	Q/A	Q/G	Q/G

^a^Q/T unique cleavage site (only found in Africa isolates)

## Discussions

Canine Kobuvirus (CaKoV) is an emerging pathogen in Thailand. To the best of our knowledge, the CaKoV was described in Asia in retrospective study in Korea in 2011 and have been reported in Japan, China and Australia, respectively [2, 15, 17, 21]. However, the CaKoV have never been reported in the country or South East Asia region. In this study, during the 2 year-survey program, we found CaKoV positivity at 17.59% in both sick (19.44%) and healthy (9.09%) animals. Compare to other studies, CaKoV % positivity in this study was lower than those in China (54%) and Korea (32.2%) [14, 22]. Our result showed that the CaKoV could be frequently detected in younger dogs at 27% which consistence with previous reports [15]. Similar to other previous studies, co-infections with other enteric viral pathogens were observed such as CaKoV/Canine parvovirus and CaKoV/Canine Coronavirus [12, 14, 15]. Moreover, CaKoVs were detected in both diarrheic and non-diarrheic dogs which consistent with other studies [2, 15]. Our result supported that this virus may not be the only cause of enteric disease in dogs. Nevertheless, the CaKoV infection have still been identified in symptomatic dogs without other enteric pathogen infections [12]. Our observation supported that the role of CaKoV as a primary pathogen of acute gastroenteritis remain unclear.

In this study, the genome size of 4 Thai CaKoVs is 7,530 bp with one ORF encoding 2,444 amino acids of a putative polyprotein, which comparable to previous reports. Genome organization of CaKoV includes leader protein (L), structural proteins (VP0, VP3, VP1), non-structural proteins (2A, 2B, 2C, 3A, 3B, 3C, 3D). Phylogenetic analyses showed that the Thai CaKoVs were closely related to each other and clustered with Aichivirus A. It is noted that Thai CaKoVs were closely related to Chinese CaKoVs sub-cluster but in separated sub-cluster from the viruses from the US, UK, Brazil and Tanzania (Fig. 3). Phylogenetic analyses of 3D gene showed similar result which Thai CaKoVs were grouped together with Chinese CaKoVs (G1 sub-cluster). This observation regarding to the sub-clusters of CaKoVs was in agreement with the previous study [23]. On the other hand, based on VP1 gene, the viruses can be clustered into 2 major subgroups, US/EU/Africa subgroup and China/Thailand subgroup which similar to the previous reports [16, 22] (Figs. 4 and 5).

Genetic analyses of Thai CaKoVs showed that whole genome of 4 Thai CaKoVs posed highest nucleotide similarity to Chinese CaKoVs including SMCD-59 and CH-1. This observation supported phylogenetic analysis that Thai CaKoVs were closely related to Chinese CaKoVs sub-cluster but in separated sub-cluster from the viruses from the US, UK, Brazil and Tanzania. Of all viral genes, the VP1 gene was the most diverse gene among Thai CaKoVs and other reference CaKoVs. Similar observation was also reported in previous study that VP1 protein is the most variable capsid protein [24]. It is noted that the putative proline rich region at VP1–228-240 (P_228_XPPPPXPPXPXP_240_) was observed both in Thai CaKoVs and reference viruses. Previous studies indicated that proline rich region may associate with enteric receptor binding of the viruses [14, 24]. It is noted that Thai CaKoVs posed unique PPP (VP1; 228–240), which also observed most reference viruses from China, Korea, Japan, US, UK suggesting unique characteristic. These unique amino acids were not observed in the CaKoV from the Australia (CE9), Brazil (BRA/26) and Tanzania (TZ/75, TZ82) [16, 20]. However, the association of these unique amino acids and viral pathogenesis is still need to be further investigated. Based on genetic analysis, unique amino acids at the position, 65 V, 67D, 119L, 138 T, 150P, 151M, 153D, 201S, 204Q, 205Q, 201Q, 213 T and 241E were observed. These unique amino acids of China/Thailand sub-cluster could be benefit for the detection of virus origin or diagnostic purpose in the future. Similar to previous study, analysis of predicted amino acid cleavage sits of whole genome were conserved among CaKoVs except one variation at 776/777 (VP3/VP1) which unique in wild carnivores [16].

## Conclusions

In conclusion, this study is the first to report of canine Kobuvirus in dogs in Thailand. CaKoVs were mostly detected in clinical dogs of young age. However, the viruses could be detected from both healthy and sicked dogs. Genetic and phylogenetic analyses showed that whole genomes of Thai CaKoVs were closely related to Chinese CaKoVs in 2015 (SMCD-59) with high nucleotide similarity suggesting a possible origin of CaKoVs in Thailand. CaKoV is considered as an emerging viral pathogen in the domestic dogs. Since CaKoVs have never been reported in the country and SEA region, the detection and characterization of CaKoV from different parts of the regions should be extended for better understanding the epidemiology and evolution of CaKoVs. Our result raises the concerns to vet practitioners that diarrhea in dogs due to canine Kobuvirus infection should not be ignored.

## Methods

### Sample collection

Sample collection was conducted in domestic dogs at small animal hospitals in Bangkok and vicinity of Thailand During September 2016 to September 2018. 307 rectal swab samples were collected from healthy dogs (*n* = 55) and dogs with gastroenteritis symptoms (*n* = 252) including vomiting, watery diarrhea, hemorrhagic diarrhea and dehydration. The swab samples were collected from dogs of young age (< 1 year) (*n* = 165), adult (1–5 years) (*n* = 98) and older (> 5 years) (*n* = 44). The animal demographic data including age, sex, breed, and vaccination history were also recorded. The ethics was conducted under the Chulalongkorn University’s animal use and care protocol # 1731074. The consent to participate of the owners of the animals used in this study was obtained in writing.

### Canine Kobuvirus (CaKoV) detection

All 307 samples were subjected to canine Kobuvirus identification by one step RT-PCR using primers specific to 3D gene of CaKoV [21]. First, RNA extraction was performed using the QIAsymphony DSP Viral/Pathogen mini kit (Qiagen, Hilden, Germany) following manufacturer’s instructions. To detect CaKoV, RNA samples were screened for 3D gene of CaKoV by using one step RT-PCR assay. The primers used in this study were previously described including U1F (5′-CATGCTCCTCGGTGGTCTCA-3′) and U1R (5′-GTCCGGGTCCATCACAGGGT -3′) [21]. Briefly, one-step RT-PCR was conducted in a total final volume of 25 μl comprising 3 μl of template RNA, 15 μl of 2xReaction Mix (Invitrogen, USA), 0.6 μl of 10 μM forward and reverse primers, 1.2 μl of SuperScript III RT (Invitrogen, USA) and distilled water to final volume 25 μl. The condition of RT-PCR assay included cDNA synthesis step at 55 °C for 30 min, next to an initial denaturation step at 94 °C for 2 min, following 40 cycles of denaturation at 94 °C for 30 s, annealing at 52 °C for 30 s and extension at 68 °C for 1 min, as well as, final extension step at 68 °C for 5 min. To confirm CaKoV, 4 μl of PCR products were run on a 1.5% agarose gel, which mixed with Red Safe at 100 V for 45 min. The expected size of CaKoV positive amplified products was 631 bp. Due to dogs showed clinical signs similar to other canine viral enteric diseases, all samples were also tested for Canine Parvovirus (*n* = 307), Canine Rotavirus (*n* = 307) and Canine Coronavirus (*n* = 30) [25–27].

### Canine Kobuvirus characterization

In this study, four CaKoV positive samples (CU-53, CU-101, CU-247 and CU-716) were selected for whole genome sequencing and additional eight CaKoV positive samples were selected for 3D and VP1 gene sequencing. The CaKoVs were selected based on epidemiological and demographic data such as age, date of isolation, breed, and vaccination history. For sequencing, nucleotide sequences of each gene of the viruses were amplified by new primer sets designed by using Primer 3 plus program [28]. List of oligonucleotide primers is provided in Additional file 1: Table S1 In brief, PCR was proceed in a final volume of 30 μl containing 2 μl of cDNA, 0.4 μM of each forward and reverse primer, 1X TopTaq Master Mix, 1X Coral Load, and distilled water. The PCR condition was set as initial denaturation at 94 °C for 3 min; 40 cycles of denaturation at 94 °C for 30 s, annealing at 50–55 °C for 45 s, extension at 72 °C for 1–1.30 min; and final extension at 72 °C for 7 min. PCR products were then purified and sequenced (1st Base Laboratories Sdn Bhd, Malaysia). Nucleotide sequences were edited, validated and assembled by using SeqMan software v.5.03 (DNASTAR Inc.; Wisconsin, USA).

### Phylogenetic and genetic analyses of canine Kobuviruses

The phylogenetic and genetic analyses were performed by comparing nucleotide sequences of Thai CaKoVs with those of Kobuvirus available from the GenBank database. The reference nucleotide sequences of CaKoVs were retrived based on their different geographic locations, host species and date of isolation. Phylogenetic analysis of CaKoV was performed by using MEGA v.6.0 (Tempe, AZ, USA) [29] with neighbor-joining method with Kimura 2-parameter with 1,000 bootstrap replicates and Beast program with Bayesian Markov chain Monte Carlo (BMCMC) with 10,000,000 generations and an average standard deviation of split frequencies < 0.05 [30]. For genetic analysis, the nucleotide sequences and deduced amino acids of CaKoV were aligned and compared using MegAlign software v.5.03 (DNASTAR Inc.; Wisconsin, USA). Pairwise comparison of nucleotides and amino acids of Thai CaKoV and those of reference CaKoVs were conducted. The variable and unique amino acids related to receptor binding of the viruses and host preferences of CaKoVs were monitored.

## Additional files

Additional file 1: **Table S1.** Oligonucleotide primers used for CaKoV whole genome sequencing. (DOCX 35 kb)

Additional file 2: **Table S2.** Association of age of CaKoVs detection in this study. (DOCX 34 kb)

## Data Availability

All data generated or analyzed during this study are included in this published article and supplement tables.

## Abbreviations

CaKoV: Canine Kobuvirus
FeKoV: Feline Kobuvirus
KoV: Kobuvirus
MuKoV: Murine Kobuvirus
