# Supplementary material for: First detection and genetic characterization of canine Kobuvirus in domestic dogs in Thailand
Source: BMC Vet Res. 2019 Jul 19;15:254. doi: 10.1186/s12917-019-1994-6 (PMC6642606; doi:10.1186/s12917-019-1994-6)
Supplement: Supplementary file 1 — Table S1. Oligonucleotide primers used for CaKoV whole genome sequencing. (DOCX 35 kb) [file 12917_2019_1994_MOESM1_ESM.docx]

**Supplement Table 1.** Oligonucleotide primers used for CaKoV whole genome sequencing

| **Primer** | **Nucleotide sequence (5’-3’)** | **Nucleotide position** |
| --- | --- | --- |
| CaKoV-1F | TGTGCCCAATCTCTTGACTCC | 10 |
| CaKoV-1R | GTGGCAATAAGGACACGGGA | 769 |
| CaKoV-2F | CCCGTGTCCTTATTGCCACT | 886 |
| CaKoV-2R | GCCTTTCCGGCGAGTTTCCC | 1558 |
| CaKoV-3F | ACAGCTCCTCAAATTCCCCG | 1181 |
| CaKoV-3R | ACAAAGGGGGAGTTCTTGGC | 2527 |
| CaKoV-4F | CACTGGAAAACCCGGATCG | 2399 |
| CaKoV-4R | GTCAGGGACAGGGATGGAG | 3273 |
| CaKoV-5F | CCTCATCCAAGGCTTCCTTT | 2974 |
| CaKoV-5R | ACTGTACTCCCACGGTTTGC | 4054 |
| CaKoV-6F | CCAATCCGGAAAAATCTGTG | 3803 |
| CaKoV-6R | ATGTGCATCAGCAAGTTTGG | 4847 |
| CaKoV-7F | ATCTCTGGACTCCTCGTCATC | 4575 |
| CaKoV-7R | GATGAGCTCGTCCAGGTTG | 5655 |
| CaKoV-8F | ACACCTCCCGAGTGATTGTC | 5394 |
| CaKoV-8R | CGTAGAGGGGGGCAGCCTTG | 6394 |
| CaKoV-9F | CCTCCAAGGCTGTCATGTCT | 6171 |
| CaKoV-9R | GTCCAGTGCACGTCGGGG | 7296 |
| CaKoV-10F | GATCCGGATTATGTCTACTCCAC | 7105 |
| CaKoV-10R | CAGTTAGAAAAGTTCAAAGACAACC | 8287 |
