# Supplementary material for: First detection and genetic characterization of canine Kobuvirus in domestic dogs in Thailand
Source: BMC Vet Res. 2019 Jul 19;15:254. doi: 10.1186/s12917-019-1994-6 (PMC6642606; doi:10.1186/s12917-019-1994-6)
Supplement: Supplementary file 2 — Table S2. Association of age of CaKoVs detection in this study. (DOCX 34 kb) [file 12917_2019_1994_MOESM2_ESM.docx]

**Supplement Table 2.** Association of age of CaKoVs detection in this study

| **Age** | **CaKoVs positive (%)** | |  |
| --- | --- | --- | --- |
|  | **Asymptomatic** | **Clinical sign** | **Total** |
| Young (< 1 year) | 2/13 (15.38%) | 40/152 (26.32%) | 42/165 (25.45%) |
| Adult (1-5 years) | 3/38 (7.89%) | 1/60 (1.67%) | 4/98 (4.08) |
| Older (>5 years) | 0/4 (0%) | 8/40 (20.00%) | 8/44 (18.18) |
|  | 5/55 (9.09%) | 49/252 (19.44%) |  |
